# Regiospecific Analysis of Fatty Acids and Calculation of Triglyceride Molecular Species in Marine Fish Oils

**DOI:** 10.1155/2018/9016840

**Published:** 2018-01-29

**Authors:** Huijun Zhang, Yingbin Shen, Youwei Zhang, Lirong Li, Xingguo Wang

**Affiliations:** ^1^Collaborative Innovation Center of Food Safety and Quality Control in Jiangsu Province, National Engineering Research Center for Functional Food, State Key Laboratory of Food Science and Technology, School of Food Science and Technology, Jiangnan University, 1800 Lihu Avenue, Wuxi, Jiangsu 214122, China; ^2^Department of Food Science and Engineering, School of Science and Engineering, Jinan University, Guangzhou, Guangdong 510632, China; ^3^School of Food Science and Technology, Jiangsu Food & Pharmaceutical Science College, 4 Meicheng Road, Huai'an, Jiangsu 223003, China; ^4^Yunnan Institute of Food Safety, Kunming University of Science and Technology, Kunming, Yunnan Province, China

## Abstract

The regiospecific distribution of fatty acids (FAs) and composition of triglyceride (TAG) molecular species of fishes were analyzed and calculated by pancreatic lipase (PL) hydrolysis and Visual Basic (VB) program. DHA was preferentially located at sn-2 position in TAG molecule, whereas EPA was almost equally distributed in each position of glycerol backbone. DOP, DPP, EPP, PoPP, PPO, and PPP were the predominant TAG species. MPP in anchovy, DDP, DOP, DPP in tuna, and EOO and OOO in salmon were the characteristic TAG molecules, which were meaningful to differentiate marine fish oils. Furthermore, the data management, according to TCN and ECN, was firstly applied to classify the TAG molecular species. The ECN42, ECN46, and ECN48 groups were rich in TAGs. The lower ECN values, compared to the higher TCN values, indicated that the most abundant TAGs exhibited a higher unsaturated degree. Therefore, our study not only offered a simple and feasible approach for the analysis of TAG composition but also firstly summarized the information by data management within ECN and TCN.

## 1. Introduction

The omega-3 long-chain polyunsaturated fatty acids (n-3 LC-PUFAs), especially cis-5,8,11,15,17-eicosapentaenoic acid (EPA) and cis-4,7,10,13,16,19-docosahexaenoic acid (DHA), are very beneficial for human health. They mainly concentrate in cell membranes and play a vital role in maintaining normal biochemical functions of the human body, such as alleviating cancer [1], cardiovascular disease [2], psychiatric disorders [3], Parkinson's disease [4], Alzheimer's Disease [5], and cardiovascular ailments [6], which has been well demonstrated in many literatures during the past three decades. In the retina and brain, DHA, concentrating in aminophospholipids of cell membranes, is as high as 95% of n-3 PUFA [7, 8]. This high concentration appears to be essential for performing optimal neuronal and retinal function [9, 10], especially as for fetuses and infants.

Additionally, EPA and DHA can be converted to other anti-inflammatory molecules, such as protectins, resolvins, maresins, and omega-3-oxylipins, which may further explain the various health benefits of omega-3 LC-PUFAs [11]. Meanwhile, EPA and DHA have been associated with many physiological functions. However, human bodies are not efficient in producing omega-3 fatty acids [12]; therefore, it is necessary to satisfy the need of our bodies through dietary. Seafood such as marine fish oils is the primary contributor of EPA and DHA. In marine fish oils, omega-3 PUFAs mainly exist in the form of triacylglycerols (TAGs) which is composed of three fatty acids esterized to a glycerol backbone with the glycerol carbons stereospecifically numbered as sn-2 (center) and sn-1,3 (outer). Different combinations of these FAs are recognized as different TAG molecular species. Therefore, the TAG molecular species composition of each oil is unique due to the complex TAG mixtures.

More importantly, the TAG composition and positional distribution of fatty acid, especially omega-3 PUFA, have strong influence on their digestion and absorption in the human bodies [13], as well as on their oxidative stability. The dietary lipids are absorbed as sn-2-(MAG) hydrolyzed on TAG by pancreatic lipase (PL) [14]. Therefore, the fatty acids in the sn-2-position of TAG were retained and absorbed during the process of digestion. The chain length, unsaturated degree, and positional distribution of fatty acids and TAG composition significantly influence their metabolism [15]. These variables reflect different nutritional value and physiological function. Additionally, the stability of PUFAs was also affected by the positional distribution and composition of fatty acids and TAG. Wijesundera et al. reported that PUFAs incorporated into the sn-2 position were susceptible to oxidation [16]. Therefore, in order to expand marine fish oils usage in food and pharmaceutical applications, it is significant to determine the composition of molecular species and positional distribution of FAs in TAG.

However, the structure and composition of TAG in natural oils are complex. Studies have depicted the characterization of different oils by using liquid chromatography mass spectrometry (LC–MS) for TAGs analysis [17–21]. Moreover, the traditional data processing is time-consuming and hard sledding; meanwhile, it is more likely to make calculating mistakes and experimental errors. According to the law of triglyceride structure and composition, a program was compiled and software was developed by using Visual Basic (VB) language to calculate the content of triglyceride composition. It can directly convert into the version of Microsoft Office Word and export the results of calculations. Furthermore, the design and operation of the software are illustrated in detail in this paper.

The purpose of the present study was not only to examine the positional distribution of fatty acids, but also to calculate the TAG composition by using VB program. The regiospecificity of TAG of marine fish oils, particularly the structure of TAG containing PUFAs such as DHA and EPA, can be obtained. Thus, this study offered an new approach to characterize the positional distribution of FAs and TAGs composition of marine fish oils rich in omega-3 PUFAs, which further improves the exploitation on the valuable underutilized source of marine fish oils.

## 2. Materials and Methods

### 2.1. Materials and Chemicals

Three marine fish oils from anchovy, tuna, and salmon were donated by Zhonghai Ocean Technology Co., Ltd. (Jiangsu, China). Silica gel GF254 thin-layer chromatography (TLC) plates were purchased from Haiyang Chemicals Co., Ltd. (Qingdao, China). All analytical and chromatographic grade chemicals were purchased from Sinopharm Chemical Reagent Co., Ltd. (Beijing, China). Standards of 40 fatty acid methyl esters (FAMEs) and PL (pancreatic lipase) were purchased from Sigma-Aldrich (St. Louis, MO, USA).

### 2.2. Triglyceride Isolation

The TAGs were isolated by thin-layer chromatography (TLC) plates with the developing solvent of hexane/diethyl ether/acetic acid (80 : 20 : 1, v/v/v). The plates were then sprayed with 0.2% 2,7-dichlorofluorescein in methanol and visualized under UV light. The band corresponding to TAG was scraped off and TAG fractions were extracted twice with 2 mL of hexane, then dried with anhydrous sodium sulfate, and concentrated with nitrogen.

### 2.3. Hydrolysis of TAG by PL (Pancreatic Lipase)

Hydrolysis of isolated TAGs from anchovy, tuna, and salmon by pancreatic lipase was carried out according to the modified method described by Luddy et al. [22]. One milliliter of 1 M Tris-HCl buffer (pH 7.8), 0.25 mL of 0.05% bile salts, 0.1 mL of 2.2% CaCl_2_, and 20 mg of pancreatic lipase were added to 50 mg of TAG. The mixture was incubated in a water bath at 37°C for 15 min with vigorous shaking, and then 1 mL of 6 M HCl was added to stop the reaction and centrifuged. The hydrolytic products were extracted three times with 2 mL of diethyl ether, then further washed twice with water to remove fatty acids, and then dried over anhydrous sodium sulfate.

### 2.4. Separation of 2-Monoacylglycerols (2-MAG) by TLC

The 2-monoacylglycerols were separated on silica gel GF254 plate with a developing solvent system comprising hexane/diethyl ether/acetic acid (50 : 50 : 1, v/v/v). The band corresponding to sn-2 MAG was scraped off and extracted twice with diethyl ether. The solvent was then removed by nitrogen and subsequently used for fatty acid analysis by GC.

### 2.5. Methylation of 2-MAG and TAG

The fatty acid methyl esters (FAMEs) of 2-MAG and TAG were prepared according to the AOCS method Ce-1b 89 (2007).

### 2.6. Gas Chromatography (GC) Analysis

The FAMEs were analyzed by a GC equipped with a flame ionization detector (Shimadzu, Tokyo, Japan) and a fused-silica capillary column (PEG-20M, 30 m × 0.32 mm × 0.5 *μ*m). The injection port and detector temperatures were both set at 250°C. The column was initially held at 80°C for 5 min, followed by temperature programming to 175°C at the rate of 15°C/min, and then held at this temperature for 5 min and programming to 215°C at the rate of 5°C/min and finally kept at this temperature for 30 min. Identification of GC peaks was finally achieved by comparing their retention times with those of the corresponding standards, and the relative contents expressed as mol% were then calculated.

### 2.7. Calculation of TAG Composition by Visual Basic (VB) Program

According to the theory of “sn-1,3 and sn-2 random” distribution of FAs in TAG, a program was performed and software was developed by Visual Basic (VB) language to calculate the TAG composition. The code of VB program is as follows:

  For i=0 To jj
  Min=i
  For j=i+1 To jj
  If ECN 0 (j) < = ECN 0 (Min)
  Then Min=j
  Next j
  t=ECN 0 (i)
  ECN 0 (i) = ECN 0 (Min)
  ECN 0 (Min)=t
  h= TAG 0 (i); TAG 0(i)
  TAG 0(i) =TAG 0(Min)
  TAG 0(Min) =h
  s= TAG 0 (i); TAG 0(i)
  TAG 0(i)=TAG 0(Min)
  TAG 0(Min) =s
  Next i

### 2.8. Statistical Analysis

All analyses were conducted in triplicate, and means ± standard deviations were calculated using Microsoft office statistical software. One-factor ANOVA and a post hoc test (Tukey-Kramer) were conducted to determine significant differences among the groups at *p* < 0.05.

## 3. Results and Discussion

### 3.1. The Overall Fatty Acid Composition in TAG

The overall fatty acid composition of TAG in the selected fish oils was shown in Table 1. As expected, DHA and EPA were the dominant PUFAs, while palmitic acid (PA) and oleic acid (OA) were the main SFAs and MUFAs. Although the fatty acids compositions are similar, significant differences of the major FAs in the content were observed between different marine fishes.

As for SFAs, the levels of C14:0 and C16:0 were significantly higher in the fish of anchovy than that in salmon and tuna, while the level of C18:0 in salmon was apparently higher than that in anchovy and tuna. For MUFAs, the most abundant FA C18:1 in salmon was detected at the highest amount in three marine fish oils, whereas C16:1 in tuna was observed to be the highest. With regard to PUFAs, the level of DHA (C22:6) was obviously the highest in tuna among the all fish species, while the content of EPA was the highest in salmon. The percentage of EPA and DHA accounted for about 20% of the total FAs and more than half of amount of the total was unsaturated fatty acid (UFA). It is well known that alpha-linolenic acid (ALA, C18:3n-3) serves as the precursor of longer chain omega-3 PUFAs and can be converted to EPA and DHA [23]. However, marine fish species are incapable of desaturating and elongating ALA (C18:3n-3) into LC-PUFAs due to their low activity of delta-6 desaturase [24]. The higher levels of PUFAs are closely related to the higher consumption of planktons which are rich in PUFAs, which finally cause the high content of EPA and DHA.

### 3.2. Positional Distribution of FAs in TAG

The positional distribution of FAs in TAG from the considered fish oils was shown in Table 2. FAs exhibited a significant difference in the regiospecific distribution among these fish species. DHA was preferentially distributed in the sn-2 position of TAG molecule, whereas EPA was almost evenly located at each position of glycerol backbone in the TAG. Herein, the even location is defined as a sn-2 regiospecificity of 33.33 mol%.

Previous studies reported that EPA showed almost equal distribution in each position of the TAG molecule, whereas DHA exhibited sn-2 positional preference on the TAG in most commercially marine fish oils. In the present study, anchovy, tuna, and salmon oils exhibited more or less similar characteristics compared to the other deep-sea fish oils manufactured as DHA and EPA supplements or nutraceuticals in food and pharmaceutical industry. The positional distribution of FAs in the TAG of anchovy was analyzed by PL hydrolysis method, and results showed 30.60% of EPA attached to sn-1,3 position, while 70.40% DHA bound to sn-2 position [25]. Akanbi et al. employed the 13C NMR spectra of major fatty acids in the anchovy oil and found EPA predominantly in positions 1 and 3, while DHA were more concentrated in position 2 [26]. In salmon oil, 28 and 57 mol% of EPA and DHA, respectively, were in sn-2 position [27]. Previous studies suggested that fatty acids were arranged and distributed in the manner of thermodynamics, which is beneficial for fish bodies to keep stable and adapt to their native environment (seawater temperature and geographical location). The preferential distribution of DHA in sn-2 positions of TAG might be more stable when marine fishes are moving in different areas at different environment temperatures [28].

Generally, the overall SFA and MUFA were preferentially presented in sn-1,3 position, but the individual SFA and MUFA displayed different positional distribution. In our study, C18:1 and C16:1 resided mostly in the terminal sn-1,3 positions, except for C16:1 in anchovy, which appeared to be evenly distributed. The residues of C18:0 and C16:0 were randomly located in sn-1,3 and sn-2 positions, except for C18:0 in anchovy, which mostly concentrated in sn-1,3 position. The positional distribution of C14:0 in each species of marine fish was different. It was mainly esterized in sn-1,3 positions in tuna; however, it showed a slight sn-2 positional preference in anchovy. Oppositely, it was equally attached to each position in salmon. Shortly, the chain length and the number of double bonds of fatty acids influenced their positional preference in the glycerol backbone of TAG molecule.

### 3.3. TAG Molecule Composition Calculated by Visual Basic (VB) Program

The TAG molecular compositions of the analyzed fish oils were calculated by using Visual Basic (VB) program based on the theory of “sn-1,3 random and sn-2 random distribution.” Table 3 illustrated the major TAG (≥1%) species and contents of the detected marine fish oils, and significant differences were observed among them. Although marine fish oils showed a wide range of TAG species, several TAG molecules were dominant in the TAG composition. Among them, the TAG molecules with the same fatty acid or three fatty acids were not abundant in marine fish oils. Most of TAG molecules contained two different fatty acids. In three marine fish oils mentioned, the combinations of fatty acids of DDP, EPP, DPP, OOO, PPO, and PPP were composed of the predominant TAG molecular species. To be specific, the FAs combinations of EOO, POO, and OOO (13.09%) were the predominant TAG molecules in anchovy, while the FAs combinations of DDP, EPP, DPP, DDP, PPO, and PPP (15.93% in anchovy and 11.93% in tuna) were the predominant TAG molecules in anchovy and tuna, respectively. Briefly, the content of each TAG species is significantly different between these marine fish oils, as well as the presence of characteristic TAG molecules. In anchovy and tuna, the predominant TAG molecules were PPP followed by PPO (7.20% in anchovy and 8.28% in tuna). It is noted that the content of MPP in anchovy was significantly higher than that of the other two fish species; thereby it can be used to distinguish marine fish oils as the characteristic TAG molecule. Similarly, DDP and DPP in tuna were apparently higher than those in anchovy and salmon as well as the characteristic TAG molecules. Differently, the largest percentage of TAG in salmon was OOO, followed by EOO and POO which were the characteristic TAG molecules distinguished to other fish species. These data confirmed that salmon contained much more oleic acids than anchovy and tuna. When comparing the other two fish species, tuna showed more complex molecular species composition, which was different from the results that the dominant TAG of the oil from belly and skeletal muscles of tuna was PDD (20.8 and 15.8%, respectively) followed by POD (9.7 and 13.0%, respectively). Our results was not consistent with previous studies which reported that, in cod liver oil, a dominant TAG was PoOP while in saury oil PED was the dominant [29]. These discrepancies may be contributed to the following factors, such as different fish species, gender, diet, season, and location.

### 3.4. TAG Group According to TCN and ECN

**Figure 1:**
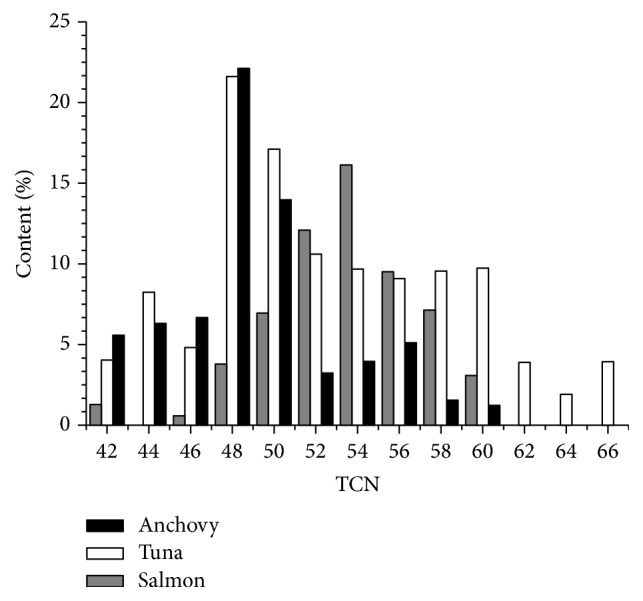
The distribution of TAGs in marine fish oils within TCN. The content% represented the percentage of TAGs within different TCN in these fish oils.

**Figure 2:**
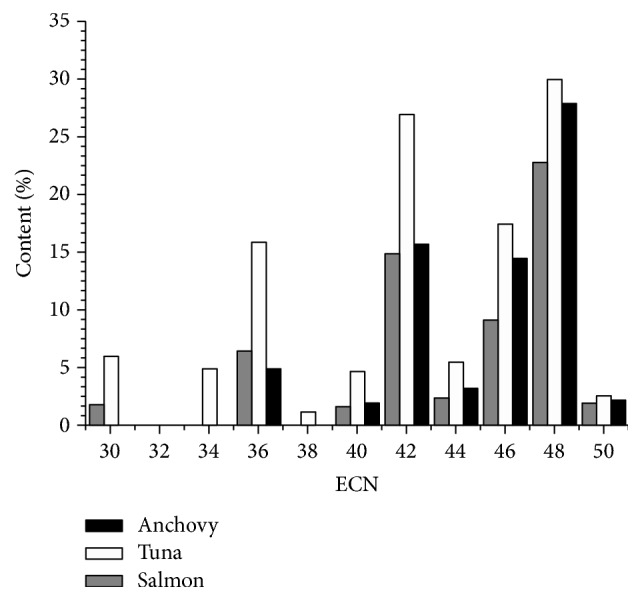
The distribution of TAGs in marine fish oils within ECN. The content% represented the percentage of TAGs within different ECN in these fish oils.

The TAG species of the evaluated anchovy, salmon, and tuna varied quite widely, as may be seen in Table 3. In order to comprehensively understand the law of FAs combinations, the TAG molecules are firstly summarized in the histograms. Figures 1 and 2 showed the TAG classes categorized by TCN (total carbon number) and ECN (equivalent carbon number), respectively. Results showed that the TAG molecular species were distributed in TCN42-66 and ECN30-50.

Specifically, the percentage of TAGs at TCN60 in tuna was apparently higher than that in the other two fish species, since tuna contained significantly higher DHA than that in anchovy and salmon. Anchovy and tuna contained a greater proportion of TAGs in TCN48 and TCN50 than salmon. Salmon and tuna in TCN52-58 contained a much higher proportion of TAGs than anchovy and the percentage of TAGs at TCN54 in salmon was the highest.

In these marine fish species, TAGs were mainly concentrated in ECN42, ECN46, and ECN48. In anchovy, these three groups mainly consisted of EPP, MPP, PPO, and PPP, which accounted for 57.92% of the total TAG. In tuna, these three bands occupied up to 74.23% of the total TAG content and DOP, DPP, EPP, PoPP, PPO, and PPP were the chiefly TAG species. In salmon, the ECN42, ECN46, and ECN48 groups were mainly composed of EOO, POO, and OOO, accounting for almost half content of the total TAG. These main TAG molecules were chiefly represented by the combinations of EPA, DHA, oleic, and palmitic acids. Briefly, the ECN48 was the maximum group accounting for almost 30% of total TAG for all three marine fish species. When comparing these marine fish species, in anchovy and tuna, PPO and PPP dominated in the TAG molecules, while in salmon OOO was the predominate TAG species.

Moreover, the TAGs distribution in each marine fish also has its own characteristics. The representative TAGs in each fish species can be used to distinguish different marine fish oils, as the relative content of them in each fish species was much significantly higher than others, such as MPP in anchovy, DDP, DOP, DPP in tuna, and EOO and OOO in salmon.

From a comparison of Figures 1 and 2, the TAG distribution within TCN and ECN presented different profiles. Results showed the lower ECN values compared with the higher TCN values, indicating that the most abundant TAGs in marine fish showed a higher unsaturated degree. The reason was that the tissues of marine fish had high concentration of polyunsaturated fatty acids, especially in liver, head, and adipose tissue.

## 4. Conclusion

In our study, the positional distributions of fatty acids and the TAG molecular species presented in anchovy, tuna, and salmon oils were analyzed, calculated, and discussed in detail. The results showed both similarities and differences among these marine fish species researched. DHA was mainly distributed in the sn-2 position, while EPA was evenly located in the sn-1,3 position of the TAG. The combinations of DOP, DPP, EPP, PoPP, PPO, and PPP were the predominant TAG species. MPP in anchovy, DDP, DOP, DPP in tuna, and EOO and OOO in salmon were the characteristic TAG molecules, which were helpful to differentiate marine fish oils. Furthermore, the data management according to TCN and ECN was firstly applied to classify the TAG molecular species. Results suggested that the ECN42, ECN46, and ECN48 groups were rich in TAGs and were mainly composed of the combination of EPA or DHA with C16:0 or C18:1. Therefore, the data management approach according to TCN and ECN of TAG molecule is useful to display the main features of each oil and generates both differences and similarities among samples to emerge. The lower ECN values compared to the higher TCN values indicated the most abundant TAGs exhibited a higher unsaturated degree. Thus, above all, these findings greatly extend the utilization of marine fish oils in food productions and may have a significant impact on the future development of the fish oil industry.

## Figures and Tables

**Table 1:** The total fatty acids composition of anchovy, tuna, and salmon.

Fatty acid	Anchovy	Tuna	Salmon
	Total	Total	Total
	(%)	(%)	(%)
C14:0	10.18 ± 0.41	8.09 ± 0.05	6.69 ± 0.14
C16:0	29.85 ± 1.21	17.09 ± 0.77	12.51 ± 0.12
C16:1	6.93 ± 0.28	10.03 ± 3.37	5.91 ± 0.82
C18:0	3.95 ± 0.16	3.39 ± 0.47	8.92 ± 0.03
C18:1	13.46 ± 0.54	18.81 ± 1.84	27.94 ± 0.69

C20:5*ω*-3	10.43 ± 0.42	9.32 ± 0.80	14.43 ± 0.57
C22:6*ω*-3	8.35 ± 0.34	18.76 ± 0.93	8.48 ± 0.70

**Table 2:** The sn-2 fatty acids composition of anchovy, tuna, and salmon.

Fatty acid	Anchovy	Tuna	Salmon
	sn-2	sn-2	sn-2
	(%)	(%)	(%)
C14:0	12.79 ± 0.59	3.12 ± 0.25	6.33 ± 0.28
C16:0	27.35 ± 1.27	16.08 ± 0.42	11.37 ± 0.66
C16:1	6.85 ± 0.32	5.06 ± 0.12	4.38 ± 1.12
C18:0	2.69 ± 0.13	3.26 ± 0.27	8.91 ± 0.64
C18:1	8.71 ± 0.40	14.68 ± 0.43	16.15 ± 1.02

C20:5*ω*-3	10.54 ± 0.63	9.56 ± 0.45	17.54 ± 0.01
C22:6*ω*-3	11.59 ± 0.54	25.88 ± 0.49	12.62 ± 1.86

**Table 3:** The species and content of TAG (>1%) in marine fish oils.

TAG species	TCN	DB	ECN	Anchovy	Tuna	Salmon
EEE	60	15	30	—	—	1.80
DDD	66	18	30	—	3.98	—
DDE	64	17	30	—	1.96	—
DDM	58	12	34	—	1.70	—
DDPo	60	13	34	—	2.11	—
DEPo	58	12	34	—	1.05	—
DDP	60	12	36	1.25	5.69^*∗*^	—
EEP	56	10	36	1.97	1.38	1.58
DEP	58	11	36	1.56	2.84	—
DDO	62	13	36	—	3.95	—
EEO	58	11	36	—	—	3.52
DEO	60	12	36	—	1.97	1.33
DPoPo	54	8	38	—	1.13	—
DMO	54	7	40	—	1.71	—
EMO	52	6	40	—	—	1.62
DMP	52	6	40	—	1.71	—
EMP	50	5	40	1.90	1.23	—
DOO	58	8	42	—	3.98	2.57
EOO	56	7	42	1.13	1.98	6.76^*∗*^
DOP	56	7	42	2.01	5.74^*∗*^	1.15
EOP	54	6	42	2.51	2.85	3.03
DPP	44	6	42	4.46	8.26^*∗*^	—
EPP	42	5	42	5.58^*∗*^	4.10	1.35
DpOO	58	7	44	—	—	1.06
MMP	44	0	44	1.86	—	—
PoMO	48	2	44	—	1.14	—
PoPoO	50	3	44	—	1.32	—
PoMP	46	1	44	1.26	1.32	—
PoPoP	48	2	44	—	1.64	—
MOO	50	2	46	1.11	1.72	3.13
MOP	48	1	46	2.45	2.47	1.40
MPP	46	0	46	5.44^*∗*^	3.56	—
PoOO	52	3	46	—	2.13	2.77
PoOP	50	2	46	1.67	3.07	—
PoPP	48	1	46	3.70	4.42	—
OOO	54	3	48	1.46	3.99	13.09^*∗∗*^
POO	52	2	48	3.24	5.75	5.86
PPO	50	1	48	7.20^*∗*^	8.28^*∗*^	2.62
PPP	48	0	48	15.96^*∗∗*^	11.93^*∗∗*^	1.18
OPS	52	1	50	—	1.04	1.87
PPS	50	0	50	2.11	1.49	—

E, EPA; D, DHA; M, myristic acid; Po, palmitoleic acid; P, palmitic acid; O, oleic acid; S, stearic acid. TCN, total carbon number; DB, double bond; ECN, equivalent carbon number. Means with different superscript letters are significantly different (^*∗*^*P* < 0.05, ^*∗∗*^*P* < 0.01).
